# Incarceration of a colonoscope in an inguinal hernia: A report of two cases

**DOI:** 10.1002/deo2.126

**Published:** 2022-05-10

**Authors:** Yuno Abe, Yuki Ohya, Osamu Nakahara, Suguru Chiyonaga, Yuto Maeda, Ryo Ichikawa, Miyuki Imamura, Satoshi Yamabe, Takeshi Morinaga, Akira Tsuji, Shintaro Hayashida, Masayoshi Iizaka, Masato Sasaki, Yukihiro Inomata

**Affiliations:** ^1^ Department of Surgery Kumamoto Rosai Hospital Kumamoto Japan; ^2^ Department of Gastroenterology and Hepatology Kumamoto Rosai Hospital Kumamoto Japan; ^3^ Department of Gastroenterological Surgery Graduate School of Medical Science, Kumamoto University Kumamoto Japan; ^4^ Department of Gastroenterology and Hepatology Graduate School of Medical Sciences, Kumamoto University Kumamoto Japan; ^5^ Department of Gastroenterology Saiseikai Kumamoto Hospital Kumamoto Japan

**Keywords:** colonoscope, inguinal hernia, sigmoid colon cancer

## Abstract

We report two cases of the rare complication of a colonoscope incarcerated in an inguinal hernia. The first patient was a 73‐year‐old man in whom a colonoscope was incarcerated in a left inguinal hernia on attempted withdrawal. The incarcerated colonoscope was successfully reduced manually under fluoroscopic guidance. The hernia was subsequently repaired using an extraperitoneal approach followed by a successful colonoscopy. The second patient was a 74‐year‐old man in whom the colonoscope became incarcerated in a left inguinal hernia on insertion. Similar to the first case, the colonoscope was manually reduced under fluoroscopy and the entire colonoscopy was then uneventfully performed. An advanced sigmoid cancer was identified and treated with sigmoidectomy. The hernia resolved after this operation. When a colonoscope becomes incarcerated in an inguinal hernia, the manual reduction should be attempted. Subsequent colonoscopy can be safely performed under certain circumstances.

## INTRODUCTION

Only a few cases of incarceration of a colonoscope in a hernia have been reported.^1^, ^2^, ^3^, ^4^ In previous reports, screening was accomplished either by subsequent colonoscopy after surgical repair of the hernia or computed tomography colonography.^2^, ^4^ Given the importance of colon cancer screening, it is critical to be able to accurately assess the colon for malignancy. Here we present two options for successfully completing the screening exam in the presence of a hernia that causes incarceration.

## CASE REPORT

### Case 1

**FIGURE 1:**
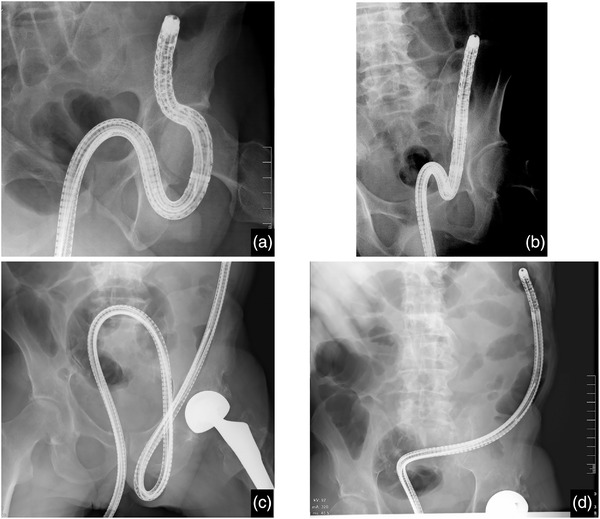
Fluoroscopy showing the incarceration of a colonoscope and colonoscope after reduction. (a,c) Fluoroscopic images of the incarcerated colonoscope in left inguinal hernia. (b) Fluoroscopic images of incarcerated colonoscope after reduction

A 73‐year‐old man underwent screening colonoscopy after several colonoscopic examinations with/without polypectomy. He had a history of hypertension, diabetes, hyperlipemia, cerebral contusion, chronic hepatitis B, and smoking. His Brinkman index was 1,800. He had not recognized the inguinal hernia before the latest colonoscopy. The procedure was initiated under mild sedation with 10 mg of diazepam and 15 mg of pentazocine administered intravenously. A PCF‐H290ZI (Olympus) colonoscope was used. After uneventful insertion to the ileocecal region and observation on the way back, the scope could not be withdrawn. Fifty centimeters of the colonoscope were left in the bowel. During the maneuver, the colonic lumen could be clearly observed. Palpation around the left inguinal region suggested the incarceration of the scope into the inguinal hernia. The patient was moved to the fluoroscopy room and sedated with 5 mg of diazepam and 15 mg of pentazocine, which were added intravenously. A loop of the colonoscope was seen in the left inguinal hernia (Figure 1a). Under direct radiographic guidance, the loop in the hernial sac was reduced by meticulous rotation, and the colonoscope was withdrawn by gentle traction and external manual assist without complication (Figure 1b). After the reduction, a colonic examination was aborted in favor of safely removing the colonoscope. The patient underwent laparoscopic totally extraperitoneal inguinal hernia repair 2 weeks after the colonoscopy.

### Case 2

**FIGURE 2:**
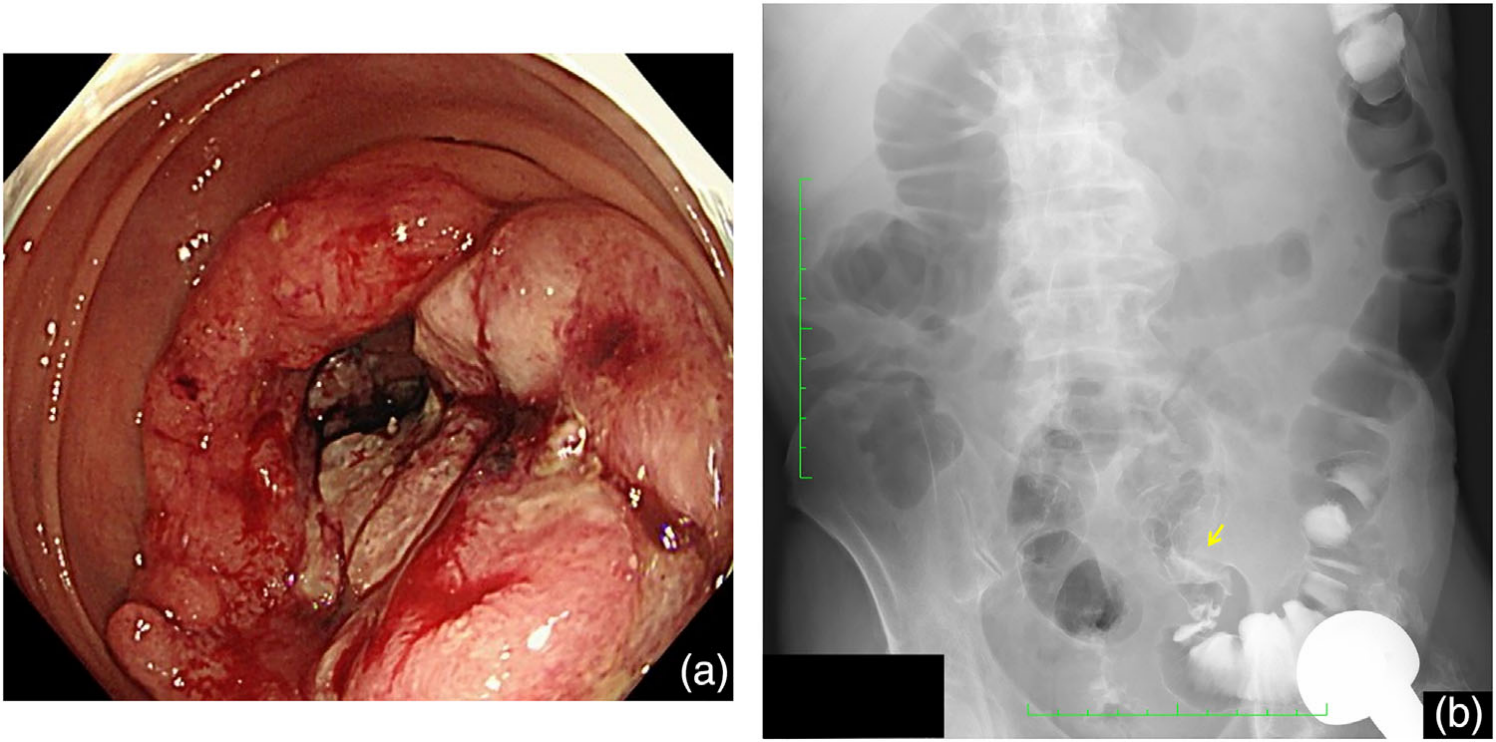
Colonoscopy image and contrast image. (a) Colonoscopy shows advanced sigmoid colon cancer. (b) Gastrografin enema shows advanced sigmoid colon cancer (yellow arrow)

A 74‐year‐old man underwent a colonoscopy for occult blood in the stool. He had a history of hypertension, diabetes, femoral neck fracture, chronic pulmonary emphysema, chronic hepatitis C, and alcoholic liver disease. He had undergone two colonoscopies. He had a history of smoking, and his Brinkman index was 800. Before the colonoscopy, he had a known left inguinal hernia, with computed tomography demonstrating the sigmoid colon in the hernia. Colonoscopy was completed under mild sedation using 4 mg of midazolam and 22.5 mg of pentazocine intravenously, and the bowel was not palpated in the inguinal region. A PCF‐H290TI (Olympus) colonoscope was used. On initial insertion to 60 cm, it became impossible to either advance the endoscope or withdraw it, although the lumen was clearly patent. A physical exam revealed bulging of the inguinal region. Incarceration of the scope was suspected, and the patient was moved to the fluoroscopy room. A loop of colonoscopes was demonstrated in the left inguinal hernia (Figure 1c). The incarcerated colonoscope was reduced under fluoroscopy by gentle traction (Figure 1d). Prior to the incarceration, an advanced sigmoid cancer was recognized making completion of the exam imperative (Figure 2). We, therefore, decided to carefully continue the colonoscopy under fluoroscopy after reduction. Reincarceration was prevented by applying external manual pressure around the groin region. No complications occurred during or after the procedure. After the colonoscopy, the patient underwent sigmoidectomy without additional examinations. After the operation, the symptom of the left inguinal hernia disappeared, probably due to the shortening of the colon and adhesion, and did not require surgery. Eighteen months after the operation, the patient has been doing well, with no tumor recurrence or symptoms of inguinal hernia.

## DISCUSSION

In both cases, the inguinal hernia was on the left side, as in many cases reported previously.^2^ In one instance, the event occurred on insertion, and in the other, on withdrawal. At the time of colonoscopy, the inguinal hernia was known in one case but not in the other. This is consistent with a previous report.^2^ In this case it was hypothesized that incarceration of the colonoscope occurred during withdrawal in a patient with an inguinal hernia with a wide hernia orifice. Because the colonoscope forms a tight loop on withdrawal, the wide hernial orifice allows the two segments of the colonoscope to enter and exit the hernia sac.^2^ Our patient 1 suffered from incarceration in a left inguinal hernia on withdrawal. This suggests that he had a wide orifice of the inguinal hernia. It is therefore surprising that he did not recognize an inguinal hernia before the colonoscopy. Risks of colonoscopy in cases with a pre‐known inguinal hernia, including cases recognized just after reduction of the incarceration, should be discussed. Due to concern for incarceration, some recommend computed tomography colonography in this setting.^2^, ^4^ In our case, we aborted the colonoscopy in one patient after reduction. Further observation was omitted because the examination was performed as a regular follow‐up without any symptoms, and there was any lesion in the colon proximal to the sigmoid. However, we continued the colonoscopy after the reduction of colonoscopy in the other patient with more concerns about abortion of the examination.

Inguinal hernia is a common disease for surgeons but not for gastroenterologists, and the incarceration of a colonoscope is very rare. Obtaining a careful history and physical examination before colonoscopy is important in identifying the possibility of this complication. If a hernia is present, postponement of colonoscopy after inguinal hernia repair should be considered. In the event the colonoscopy must be performed prior to hernia repair, fluoroscopic guidance is recommended to prevent the incarceration of a colonoscope. External manual pressure during colonoscopy may be possible and effective. If the colonoscope cannot be inserted or removed with the lumen visible during the examination, incarceration of a colonoscope should be suspected and confirmed on examination. Continued manipulation may worsen the incarceration or cause perforation. Safe reduction under fluoroscopic guidance should be performed as soon as possible with surgical consultation.^1^, ^2^, ^3^, ^4^ Fluoroscopic guidance and simultaneous gentle manual pressure to reduce the incarcerated colonoscope through the hernial orifice are recommended.^1^, ^2^, ^3^, ^4^ If this method does not work, a “pulley” method needs to be tried. If this is unsuccessful, surgery needs to be performed.

As in our two patients, past uneventful colonoscopy could not completely exclude the possibility of inguinal hernia. Older age, male sex, smoking, protease inhibitors, chronic emphysema, and so forth, are known risk factors for inguinal hernia.^5^, ^6^ Our patients had three or more of the risk factors. It is important to pay attention to a patient who has risk factors even if the patient did not have a history of inguinal hernia.

In conclusion, when a colonoscope cannot be inserted or removed with the lumen visible during colonoscopy, incarceration of a colonoscope into an inguinal hernia should be suspected. Palpation and fluoroscopic examination of the inguinal region should be performed to establish the diagnosis and prevent perforation or other complications. Subsequent colonoscopy after manual reduction may be acceptable in selected patients.

## CONFLICT OF INTEREST

The authors declare no conflict of interest.

## FUNDING INFORMATION

None.
